# Intranasal Administration of Bedaquiline-Loaded Fucosylated Liposomes Provides Anti-Tubercular Activity while Reducing the Potential for Systemic Side Effects

**DOI:** 10.1021/acsinfecdis.4c00192

**Published:** 2024-08-13

**Authors:** Franziska Marwitz, Gabriela Hädrich, Natalja Redinger, Karen F. W. Besecke, Feng Li, Nadine Aboutara, Simone Thomsen, Michaela Cohrs, Paul Robert Neumann, Henrike Lucas, Julia Kollan, Constantin Hozsa, Robert K. Gieseler, Dominik Schwudke, Marcus Furch, Ulrich Schaible, Lea Ann Dailey

**Affiliations:** † Bioanalytical Chemistry, Research Center Borstel, Leibniz Lung Center, Parkallee 1−40, Borstel 23845, Germany; ‡ German Center for Infection Research, Thematic Translational Unit Tuberculosis, Borstel 23845, Germany; § Department of Pharmaceutical Sciences, University of Vienna, Josef-Holaubek-Platz 2 ,Vienna 1090, Austria; ∥ Institute of Pharmacy, Martin-Luther-Universität Halle-Wittenberg, Kurt-Mothes-Str. 3, Halle/Saale 06120, Germany; ⊥ Cellular Microbiology, Research Center Borstel, Leibniz Lung Center, Parkallee 1−40 ,Borstel 23845, Germany; # Rodos Biotarget GmbH, Feodor-Lynen-Straße 31, Hannover 30625, Germany; ∇ Siegfried Hameln GmbH, Langes Feld 13 ,Hameln 31789, Germany; ○ Cardior Pharmaceuticals GmbH, Hollerithallee 20 ,Hannover 30419, Germany; ◆ Vienna Doctoral School of Pharmaceutical, Nutritional and Sport Sciences (PhaNuSpo), University of Vienna, Josef-Holaubek-Platz 2 ,Vienna 1090, Austria; ¶ General Biochemistry and Physical Pharmacy, Faculty of Pharmaceutical Sciences, Ghent University, Ottergemsesteenweg 460 ,Ghent 9000, Belgium; & Department of Medicine, University Hospital, Knappschaftskrankenhaus Bochum, Ruhr University Bochum, In der Schornau 23−25 ,Bochum 44892, Germany; ● German Center for Lung Research (DZL), Airway Research Center North (ARCN), Research Center Borstel, Leibniz Lung Center, Borstel 23845, Germany; ◊ Kiel Nano, Surface and Interface Sciences (KiNSIS), Kiel University, Kiel 24118, Germany; ▲ Certmedica International GmbH, Magnolienweg 17 ,Aschaffenburg 63741, Germany

**Keywords:** bedaquiline, liposomes, inhalation, tuberculosis, pharmacokinetics

## Abstract

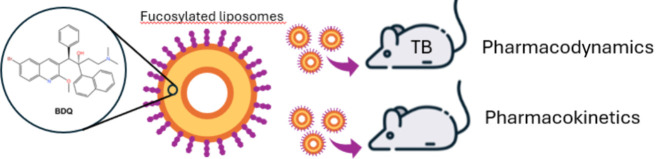

Liposomal formulations of antibiotics for inhalation offer the potential for the delivery of high drug doses, controlled drug release kinetics in the lung, and an excellent safety profile. In this study, we evaluated the *in vivo* performance of a liposomal formulation for the poorly soluble, antituberculosis agent, bedaquiline. Bedaquiline was encapsulated within monodisperse liposomes of ∼70 nm at a relatively high drug concentration (∼3.6 mg/mL). Formulations with or without fucose residues, which bind to C-type lectin receptors and mediate a preferential binding to macrophage mannose receptor, were prepared, and efficacy was assessed in an *in vivo* C3HeB/FeJ mouse model of tuberculosis infection (H37Rv strain). Seven intranasal instillations of 5 mg/kg bedaquiline formulations administered every second day resulted in a significant reduction in lung burden (∼0.4–0.6 Δlog_10_ CFU), although no differences between fucosylated and nonfucosylated formulations were observed. A pharmacokinetic study in healthy, noninfected Balb/c mice demonstrated that intranasal administration of a single dose of 2.5 mg/kg bedaquiline liposomal formulation (fucosylated) improved the lung bioavailability 6-fold compared to intravenous administration of the same formulation at the same dose. Importantly, intranasal administration reduced systemic concentrations of the primary metabolite, *N*-desmethyl-bedaquiline (M2), compared with both intravenous and oral administration. This is a clinically relevant finding as the M2 metabolite is associated with a higher risk of QT-prolongation in predisposed patients. The results clearly demonstrate that a bedaquiline liposomal inhalation suspension may show enhanced antitubercular activity in the lung while reducing systemic side effects, thus meriting further nonclinical investigation.

Bedaquiline (BDQ; previously referred to as TMC-207 or R027910) is a diarylquinoline antimycobacterial agent approved in 2012 (USA)^1^/2014 (Europe) as a part of a multidrug treatment regimen for pulmonary multidrug-resistant tuberculosis (MDR-TB;^2^). This first-in-class compound inhibits ATP synthase in the mycobacteria with a high selectivity, i.e., showing a >20,000 higher affinity for mycobacterial ATP synthase versus eukaryotic ATP synthase.^3^ It is marketed by Janssen-Cilag under the brand name Sirturo as an uncoated immediate release tablet (100 mg free base) for oral administration, whereby the typical dosing regimen consists of 400 mg daily for the first 2 weeks followed by 200 mg thrice weekly for 22 consecutive weeks as part of a combination antituberculous treatment regimen.^4^

BDQ is practically insoluble in aqueous media (estimated: 0.002 μg/mL at 25 °C with an estimated log*P* of 7.74).^5^ When administered orally, BDQ shows a high bioavailability with a median *t*_max_ value of ∼5 h.^6^ As a lipophilic compound, BDQ absorption is influenced by food intake, whereby coadministration with high-fat meals can increase both the *C*_max_ and AUC by 2-fold. Its extreme lipophilicity results in an extensive accumulation in peripheral tissues (volume of distribution = 164 L; > 99% protein binding), a triexponential elimination profile, and a terminal elimination half-life of around 4–5 months.

The high tissue accumulation results not only from the lipophilicity of the drug but also from its cationic amphiphilic nature. So-called cationic amphiphilic drugs (CADs) are known to bind to phospholipids resulting in intracellular accumulation in cells and tissues, the generation of phospholipid inclusion bodies also known as drug-induced phospholipidosis (DIPL).^7,8^ BDQ is metabolized by CYP3A4 into its major metabolite, N-desmethyl bedaquiline (M2; Figure 1), which retains an antimycobacterial activity (4–6-fold lower than BDQ), as well as CAD properties.^6^ Although the mechanisms are still not fully understood, systemic CAD exposure and DIPL are associated with inhibition of the potassium ion channel encoded by the human ether-à-go-go-related gene (hERG). Inhibition of hERG channels results in QT interval prolongation, which can result in life-threatening ventricular tachyarrhythmia.^9,10^ Clinical studies with oral BDQ have shown mild but significant increases in QT prolongation in treated cohorts compared with placebo, which was generally reversible following termination of the treatment. Co-administration of BDQ with other drugs causing QT prolongation, such as fluoroquinolones and clofazimine, revealed an additive effect, resulting in a recommendation issued by the World Health Organization to restrict coadministration of compounds with known QT prolongation to TB control programs that provide QT interval monitoring.^11^ The M2 metabolite has been shown *in vitro* to cause a higher cytotoxicity and phospholipidogenesis.^12,13^ It is postulated that M2 levels may therefore be more strongly associated with QT prolongation compared to the parent compound BDQ.^13^

**Figure 1:**
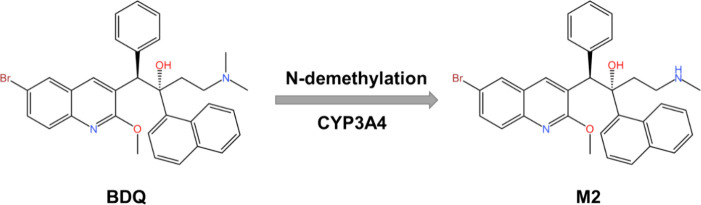
BDQ is metabolized by CYP3A4 to N-desmethyl-BDQ, also known as M2.^14^

Pulmonary administration of antitubercular agents offers the potential to achieve higher local drug concentrations in the lung at the site of infection while overall reducing systemic adverse effects.^15,16^ In the case of BDQ, inhalation administration might reduce systemic BDQ/M2 concentration ratios, thereby achieving a reduced incidence of QT prolongation. As a consequence, the scope for coadministration of bedaquiline with other therapeutic agents might be significantly broadened. Unfortunately, the poor aqueous solubility of BDQ causes challenges for pulmonary delivery. There is increasing evidence that inhaled dry powders of poorly soluble compounds are associated with adverse effects in the lung, including particulate accumulation, increased macrophage numbers, increased prevalence of foamy macrophages, and particle-induced inflammation.^17,18^ To circumvent these issues, a liposomal delivery system^19,20^ was developed to encapsulate therapeutically relevant concentrations of BDQ for administration as a stable liquid nanodispersion. A subset of the BDQ-loaded liposomes was functionalized with fucosyl residues, which bind to C-type lectin receptors (CLR) and mediate a preferential binding to the macrophage mannose receptor (CD206) and DC-SIGN (CD209) present on human alveolar macrophages.^19^ Targeting alveolar macrophages may confer some therapeutic benefits, especially during the early stages of *Mycobacterium tuberculosis* (Mtb) infection and dissemination,^21,22^ since phagocytized *Mtb* is capable of avoiding lysosomal acidification and evading immune responses.^23−25^

In the current study, the pharmacodynamic activity of BDQ-loaded fucosylated/nonfucosylated liposomes (BDQ-Lipo_fuc_/BDQ-Lipo) was investigated in a mouse model of *Mtb* infection, which forms caseating necrotic granulomas and thus more closely resembles the human pathology.^26,27^ In addition, a comparative study of BDQ pharmacokinetics (PK) of intranasally and intravenously administered BDQ-Lipo_fuc_ versus orally administered BDQ powder (neat drug) was used to assess whether alternative delivery routes can increase lung concentrations of BDQ, while simultaneously decreasing systemic exposure to the major metabolite, M2, which is correlated with the problematic side-effect of QT-prolongation.^13^

## Results and Discussion

### Properties of the Bedaquiline-Loaded Liposomal Systems

The liposomal formulations with and without CLR-targeting function used in the current study were developed by the company Rodos Biotarget GmbH and belong to a technology platform marketed under the name TargoSpheres.^19^ The TargoSphere platform has been shown to successfully encapsulate levofloxacin and BDQ with encapsulation efficiencies of 66–80 or ∼98%, respectively.^20^ The BDQ loading capacity was 5–7% of the total mass.^20^ In the current study, we confirmed the reported characterization data with an analysis of six further independent batches (Table 1). Cryo-electron microscopy images from Huck et al.^20^ depict BDQ-Lipo_fuc_ systems as small (50–100 nm), predominantly unilamellar systems with a subfraction of multilamellar vesicles. Dynamic light scattering (DLS) size measurements from the current study confirmed this observation. It is further notable that BDQ encapsulation generally increased the mean Z-Ave values of the nonmodified liposomes (BDQ-Lipo), indicating a possible destabilization of the lipid membrane caused by the drug. Fucosylated systems (BDQ-Lipo_fuc_) showed a much lower size and polydispersity, possibly due to enhanced colloidal stability of the liposomes via steric hindrance mechanisms.

**Table 1:** Formulation Properties (Mean ± Standard Deviation from *n* = 6 Independent Batches)^a^

formulation	study employed in	BDQ content (mg/mL)	lipid content (mg/mL)	particle size (nm or μm)	PDI
BDQ-Lipo_fuc_	*in vitro*, PK, PD	3.58 ± 0.44	∼64	70 ± 2 nm	0.109 ± 0.057
Lipo_fuc_	*in vitro*, PK, PD	NA	∼64	66 ± 3 nm	0.055 ± 0.011
BDQ-Lipo	*in vitro*, PK, PD	3.01 ± 0.55	∼64	121 ± 43 nm	0.353 ± 0.194
Lipo	*in vitro*, PK, PD	NA	∼64	70 ± 1 nm	0.048 ± 0.010
BDQ neat	PK only	4	0	23 ± 4 μm	NA
BDQ solution (HPCD)	PD only	3.6	0	NA	NA
BDQ (DMF dilution)	*in vitro* only	0.001–0.00001	0	ND	NA

a NA = not applicable; ND = not determined; HPCD = 2-hydroxypropyl-β-cyclodextrin.

Despite the low drug loading capacity, the TargoSphere liposomal formulations are stable at a relatively high concentration equating to ∼3.5 mg/mL BDQ and ∼64 mg/mL total lipids with a lipid:drug weight ratio of 0.94. In comparison, the commercial product, amikacin liposome inhalation suspension (Arikayce), comprising dipalmitoylphosphatidyl choline (DPPC)/cholesterol liposomes, has a lipid:drug weight ratio of 0.60–0.79. Calculated from a single amikacin dose (590 mg per 8.4 mL vial), the Arikayce lipid concentration ranges from 112 to 126 mg/mL per vial. This comparison provides two important points of reference. The first is that the amount of lipid excipient in the BDQ-Lipo_fuc_ formulation is approximately 50% lower than in the Arikayce product and therefore likely to be well tolerated in the lung. The second point is that a single 10 mL dose of nebulized BDQ-Lipo_fuc_ formulation could administer 40 mg of BDQ or 10% of the recommended oral daily starting dose and 20% of the maintenance dose.

The high doses achievable by a nebulized liposomal suspension can provide advantages over dry powder formulations for highly lipophilic compounds such as BDQ with poor aqueous solubility. Although there are respirable dry powders comprised predominantly of the active pharmaceutical ingredient (API) which have been engineered to deliver high API doses (e.g., Inbrija with 42 mg levodopa per capsule^28^ and TOBI with 28 mg tobramycin per capsule^29^), the spray-drying methods used to produce these powders typically require APIs with a high aqueous solubility. Spray-dried liposomes^30^ or nanoemulsions^31^ have been used to generate respirable dry powders for hydrophobic drugs, but achieving high drug content per mg powder remains challenging and is highly dependent on API properties. For example, the spray-dried powders containing BDQ-loaded liposomes investigated by Huck et al. (2022) achieved only ∼1 μg BDQ per mg powder.^20^

### *In Vitro* and *In Vivo* Efficacy of Liposomal BDQ Formulations

**Figure 2:**
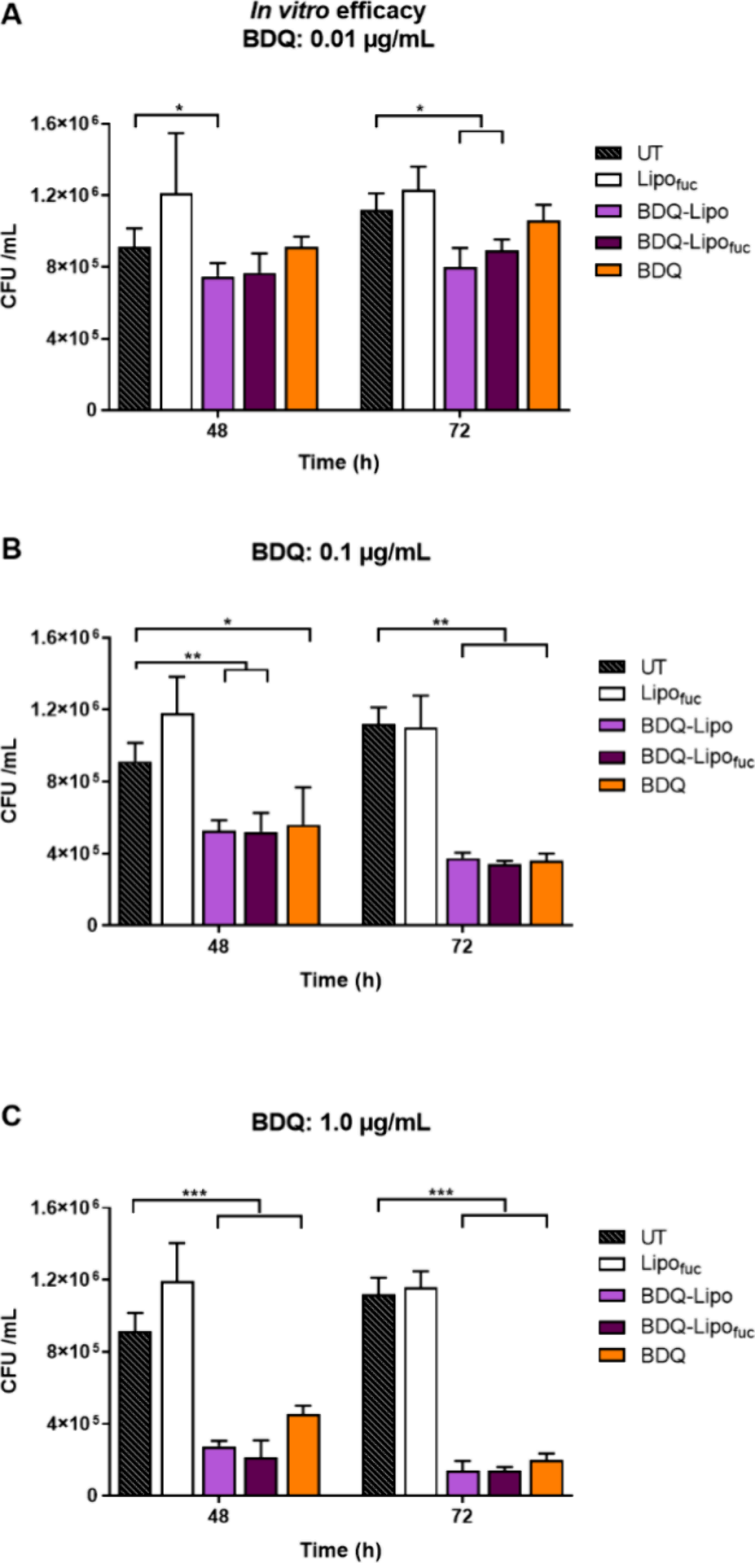
In vitro activity of fucosylated and nonfucosylated BDQ-Lipo formulations compared to nonformulated BDQ at doses of (A) 0.01, (B) 0.1, and (C) 1 μg/mL over 48 and 72 h. Values represent the mean ± standard deviation of triplicate experiments. *p < 0.05; **p < 0.01; ***p < 0.001.

The minimum inhibitory concentration (MIC) and minimum bactericidal concentration (MBC) of BDQ against the susceptible H37Rv strain of *Mtb* are reported as 0.06 and 0.3 μg/mL, respectively.^12^ In an *in vitro* model of primary BMDM infected with the H37Rv strain, nonformulated BDQ (dissolved in DMF then diluted in cell culture medium) was used as a positive control and achieved significant reductions in CFU/mL above the reported MIC at the doses 1 and 0.1 μg/mL (Figure 2). In contrast, both BDQ-Lipo formulations (with and without fucosylation) significantly reduced CFU/mL, even at 0.01 μg/mL (Figure 2). It may be feasible that the liposomal formulation improves both cellular uptake and availability of BDQ in the in vitro setting. This becomes especially prominent at the lowest dose, where higher intracellular drug concentrations can already have an effect on mycobacterial counts. However, a statistical analysis using ANOVA with Dunnett’s correction for multiple comparisons shows that the liposomal formulations were only significantly better than the unformulated BDQ in two groups: BDQ vs BDQ-Lipo, 0.01 mg/mL for 72 h incubation (*p* = 0.0054) and BDQ vs BDQ-Lipo_fuc_ 1.0 mg/mL for 48 h incubation (*p* = 0.0002). Based on a lack of consistent statistical differences, we decided that it may be too speculative to claim that liposomal uptake improves antibacterial performance.

The fucosylated liposomal formulation did not show significantly higher antimycobacterial activity *in vitro*. This was unexpected since *Mtb* infection of BMDM has been shown to induce macrophage polarization toward an M2 phenotype with concurrent increases in CD206 expression.^32^ However, *in vitro* studies evaluating CD206 expression in *Mtb*-infected murine BMDM report that only a fraction of the cell population (∼15%; Zhang, 2020;^32^ and ∼25%; Wang, 2013^33^) expressed CD206 under the conditions tested, which might explain why a targeting enhancement by fucosylated liposomes was not detectable in this experiment. Furthermore, Durán et al. (2021) reported that a preferential uptake of fucosylated liposomal formulations was only observed in human dendritic cells and monocytes but not in human interstitial and alveolar macrophages. They hypothesized that the high phagocytosis capacity of macrophages may play a more prominent role in cellular uptake compared to the CLR targeting effect in this cell type.^19^

**Figure 3:**
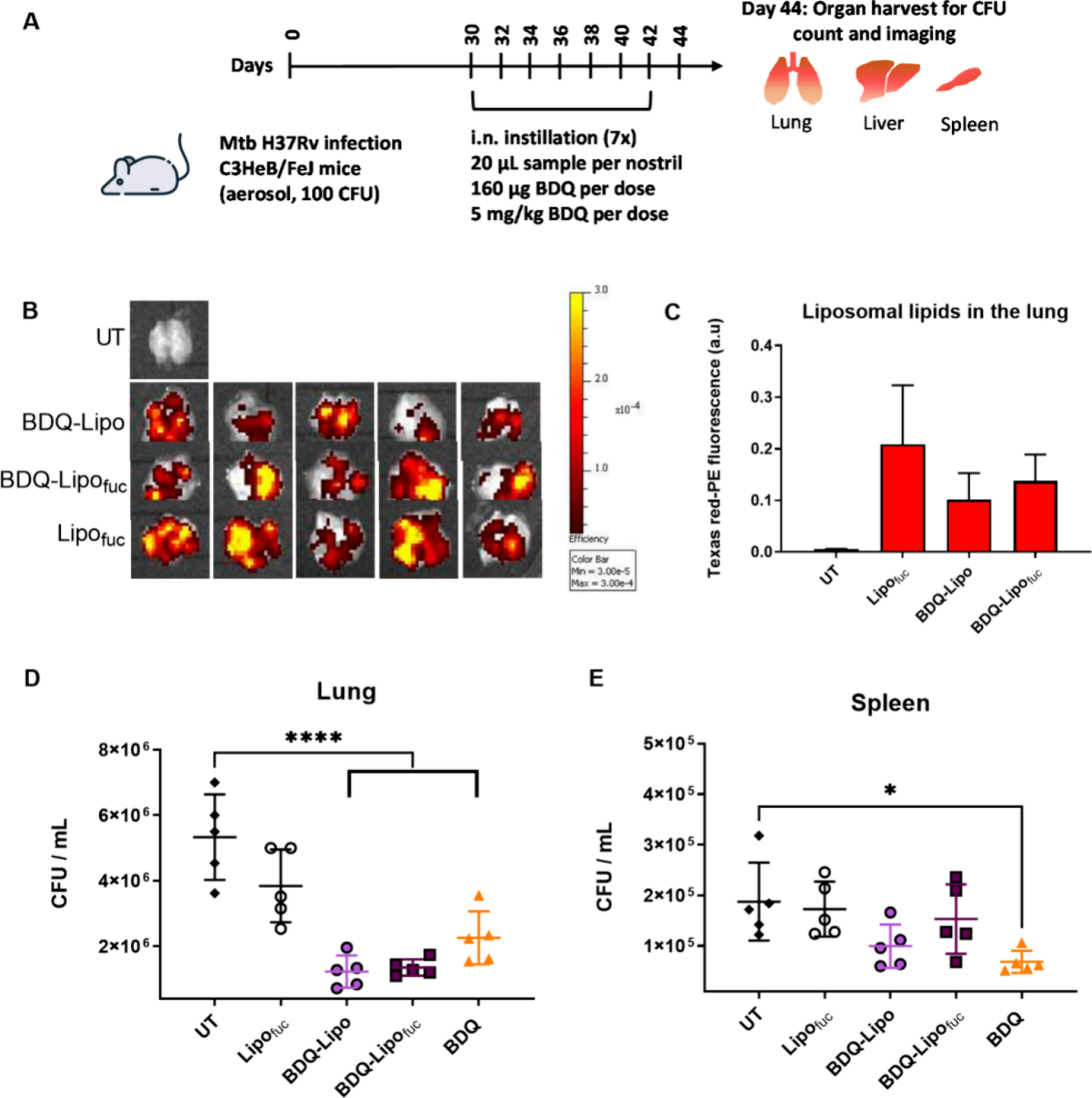
(A) Schematic of in vivo antitubercular activity assessment of fucosylated and nonfucosylated BDQ-Lipo formulations compared to nonformulated BDQ in Mtb infected C3HeB/FeJ mice following seven i.n. instillations every second day for 14 days. BDQ formulations contained 160 μg of BDQ (5 mg/kg) per administration, and the unloaded Lipo_fuc_ formulation was used as a vehicle control. Texas Red-PE was incorporated into the liposomal formulations to assess variability of liposomal content in the lung on day 44 (B) enabling semiquantitative assessment of fluorescence intensity from *n* = 5 lungs (C). CFU/organ in lung (D) and spleen (E) were determined from organ homogenates. Values represent the mean ± standard deviation of *n* = 5 animals per treatment group. UT = Untreated Mtb-infected control group. *p < 0.05; ****p < 0.0001.

The same formulations were tested for antitubercular activity in a murine model of TB following i.n. administration of 20 μL sample per nostril every second day for 7 days equating to a nominal dose of 5 mg/kg BDQ per administration (Figure 3A). Here, we chose to administer the undiluted liposomal formulations with the aim of maximizing the therapeutic dose achievable via intranasal administration in this mouse model. Using the data reported by Southam et al. (2002)^34^ to estimate the biodistribution of radiolabeled colloids following i.n. instillation, ∼40% of a 20 μL volume instilled intranasally will be aspirated into the lung, while the remaining 60% drains from the nasal cavity into the gastrointestinal (GI) tract. Drug absorption into the systemic circulation may therefore occur in the nasal passages, the lungs, and the GI tract. Thus, the contribution of lung dose toward pharmacodynamic activity cannot be fully separated from systemic dose via nasal and GI absorption in this study.

Using Texas Red-PE-labeled liposomes, it was possible to provide a semiquantitative comparison of residual liposomal components in the lung at the end of the treatment regimen (Figure 3B,C) demonstrating indirectly that approximately equal amounts of formulation reached the lung using this administration technique and a fairly homogeneous lipid distribution in lung tissue was observed (Figure 3B and Figure S1). Assessment of liposomal lipid and BDQ colocalization within the lung was not possible within the scope of this experiment.

All BDQ treatment groups significantly reduced *Mtb* CFU/mL counts in the lung compared to the untreated and liposomal vehicle control (Figure 3D; *p* < 0.0001). The relative reduction in lung burden (Δlog_10_ CFU/mL) following seven intranasal administrations was 0.38, 0.65, and 0.59 for BDQ (HPCD), BDQ-Lipo and BDQ-Lipo_fuc,_ formulations, respectively. One way ANOVA using Tukey’s multiple comparison test showed no significant differences between the efficacy of fucosylated and nonfucosylated liposomal formulations (*p* = 0.9362). Compared to the solubilized BDQ treatment group (HPCD), the performance of the BDQ-Lipo formulation was significantly improved (*p* = 0.0339). While the performance of BDQ-Lipo_fuc_ was not significantly better than that of the solubilized drug (*p* = 0.0626), the results also showed a trend in this direction. The solubilized BDQ (HPCD) was the only formulation to show a significant reduction in CFU counts in the spleen (Figure 3E; *p* < 0.05). The overall results may indicate a more rapid permeability of presolubilized BDQ (using cyclodextrins as solubilization agents) across the air-blood barrier resulting in a slightly lower lung exposure but higher systemic exposure. Conversely, the liposomal system may help retain BDQ in the lung, possibly improving the pulmonary antitubercular efficacy, although longer study trials would be required to confirm this hypothesis. Similar to the *in vitro* results, fucosylation of the liposomes did not enhance therapeutic performance in this murine infection model, as has been previously hypothesized.^19^ Instead, drug and liposomal properties appear to be more influential in this disease model.

### Comparative Pharmacokinetics: Intranasal Versus Intravenous and Oral Administration

**Figure 4:**
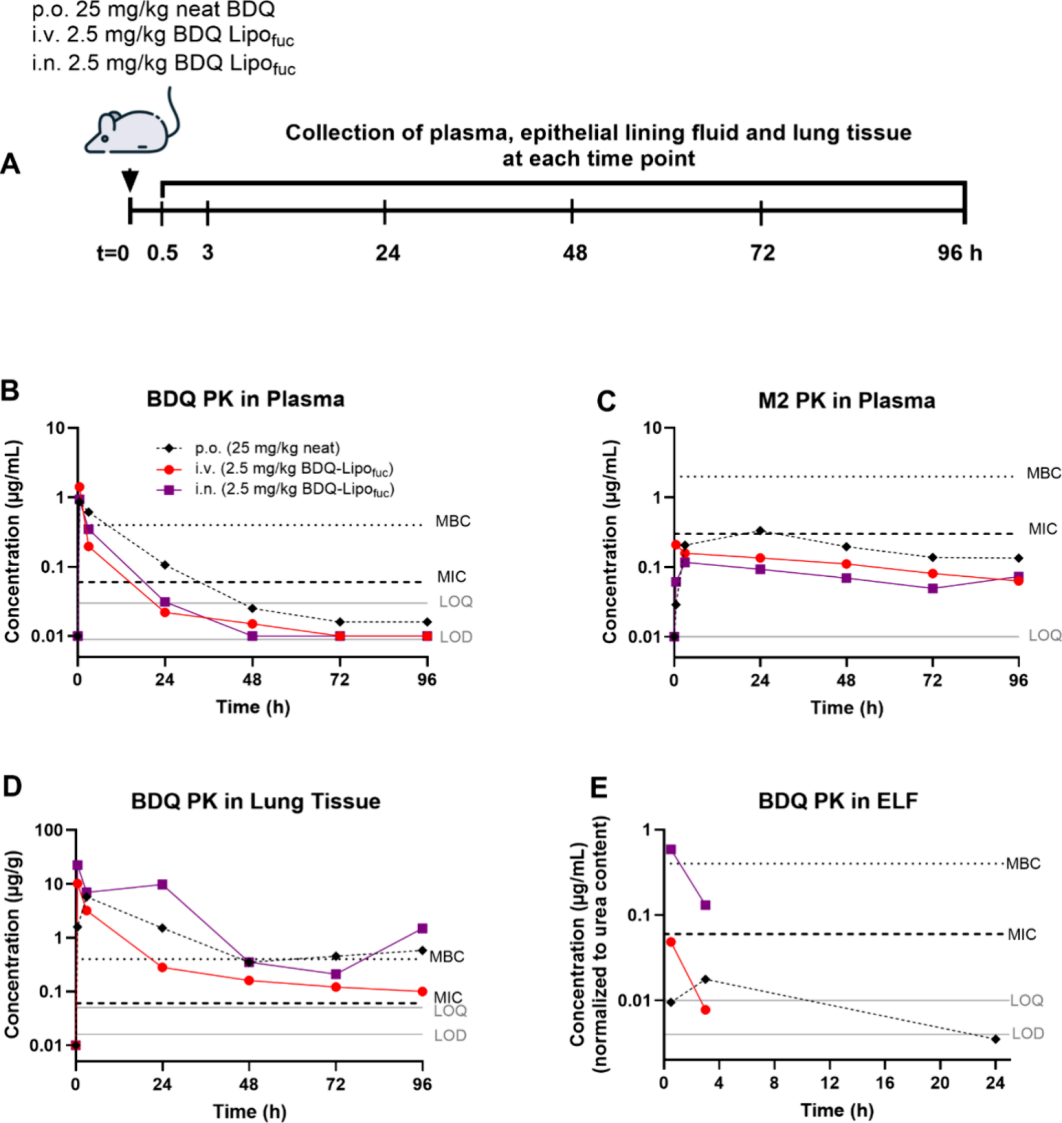
(A) Schematic of the PK study comparing i.v. and i.n. administration of BDQ-Lipo_fuc_ formulations to oral administration of neat BDQ. (B) BDQ and (C) M2 plasma concentrations over 96 h were determined and compared with BDQ concentrations in (D) lung tissue and (E) ELF. Values represent the mean of six animals per time point. Individual replicate values are depicted in the Supporting Information Figure S2. MBC and MIC values for BDQ and M2, depicted as dotted lines, were cited from Rouan et al.^12^ Gray lines depict the LOQ and LOD for each individual compartment.

To investigate whether i.n. administration of BDQ-Lipo_fuc_ does achieve higher BDQ lung concentrations compared with the oral administration route, a comparative PK study was conducted. To better reflect the conventional standard-of-care product (tablet as a dosage form), BDQ was administered via oral gavage as a drug suspension with a mean particle size of 23 ± 4 μm. An oral dose of 25 mg/kg was chosen for direct comparison to Irwin et al. (2016), with the notable difference that Irwin et al. used HPCD to solubilize BDQ as an inclusion complex prior to administration.^27^ A 10-fold lower dose of 2.5 mg/kg was chosen for the i.n. administration route in the PK evaluation. Since it was possible to safely administer the BDQ-Lipo_fuc_ formulation via i.v. administration, an i.v. treatment group (2.5 mg/kg) was also included in the study, with the understanding that the CLR-targeted liposomal formulation will likely influence the PK profile of the BDQ compared to nonformulated API. Healthy, noninfected Balb/c mice were administered a single dose via p.o., i.n. or i.v. administration and one group (*n* = 3 male, *n* = 3 female) of animals were culled per time point (0.5, 3, 24, 48, 72, and 96 h) to quantify BDQ in plasma, lung tissue homogenate, epithelial lining fluid (ELF), and the cellular fraction of the broncho-alveolar lavage using LC-MS/MS (Figure 4A; Figure S2).

BDQ and M2 concentrations quantified in plasma (Figure 4B,C) were generally comparable to values reported by Rouan et al. (2012)^12^ and Irwin et al. (2016)^27^ providing confirmation that the methodology employed in the current study was robust. A comparison of the noncompartmental PK parameters in plasma (Table 2) revealed that the oral administration group in the current study exhibited a lower overall *C*_max_ and AUC compared to orally administered BDQ from both the Rouan et al.^12^ and Irwin et al. studies. This was expected since administration of a BDQ neat drug suspension will necessarily involve an additional dissolution phase in the GI tract, which is not the case for BDQ solubilized with cyclodextrins, which may alter bioavailability compared to cyclodextrin-based formulations.

**Table 2:** Noncompartmental PK Data in Plasma and Lung Tissue, Comparing the Results of the Current Study to Previously Published Data

**BDQ: plasma**						
**study**	**route**	**dose** (mg/kg)	**dosage form**	*C*_**max**_**(μg/mL)**	*t*_**max**_**(h)**	**AUC** (μg h/mL)
Rouan et al.^12^	p.o.	30	HPCD solution	2.1	3	26.3 (0–168 h)
Irwin et al.^27^	p.o.	25	HPCD solution	2.9	0.5	33.7 (0–168 h)
current	p.o.	25	drug suspension	0.9	0.5	11.4 (0–96 h)
current	i.v.	2.5	BDQ-Lipo_fuc_	2.1		4.2 (0–96 h)
current	i.n.	2.5	BDQ-Lipo_fuc_	0.9	0.5	5.8 (0–96 h)
**M2: plasma**						
**study**	**route**	**dose** (mg/kg)	**dosage form**	*C*_**max**_**(μg/mL)**	*t*_**max**_**(h)**	**AUC** (μg h/mL)
Rouan et al.^12^	p.o.	30	HPCD solution	1.0	24	71.5 (0–168 h)
Irwin et al.^27^	p.o.	25	HPCD solution	0.8	8	57.3 (0–168 h)
current	p.o.	25	drug suspension	0.3	24	18.7 (0–96 h)
current	i.v.	2.5	BDQ-Lipo_fuc_	0.1	0.5	9.6 (0–96 h)
current	i.n.	2.5	BDQ-Lipo_fuc_	0.2	3	6.3 (0–96 h)
**BDQ: lung tissue**						
**study**	**route**	**dose** (mg/kg)	**dosage form**	*C*_**max**_**(μg/mL)**	*t*_**max**_**(h)**	**AUC** (μg h/mL)
Irwin et al.^27^	p.o.	25	HPCD solution	22.1	8	694.0 (0–168 h)
current	p.o.	25	drug suspension	5.7	3	124.7 (0–96 h)
current	i.v.	2.5	BDQ-Lipo_fuc_	10.2	0.5	62.2 (0–96 h)
current	i.n.	2.5	BDQ-Lipo_fuc_	22.3	0.5	360.2 (0–96 h)
**BDQ: ELF**						
**study**	**route**	**dose** (mg/kg)	**dosage form**	*C*_**max**_**(μg/mL)**	*t*_**max**_**(h)**	**AUC** (μg h/mL)
current	p.o.	25	drug suspension	0.02	3	0.08 (0–3 h)
current	i.v.	2.5	BDQ-Lipo_fuc_	0.05	0.5	0.05 (0–3 h)
current	i.n.	2.5	BDQ-Lipo_fuc_	0.59	0.5	1.01 (0–3 h)

Interestingly, i.n. administration resulted in marginally higher BDQ and lower M2 concentrations in plasma compared to i.v. administration of the same dose of liposomal formulations (Figure 4B,C). Concentrations of BDQ in tissue homogenates of lavaged lungs showed substantially higher amounts of BDQ following i.n. administration. The increase observed in drug concentration in the lung tissue at 96 h for i.n. administered BDQ liposomes was unexpected. We hypothesize that this might be due to the unusual partitioning behavior of BDQ in the body over time, although we cannot confirm this directly. BDQ is reported to exhibit triphasic elimination kinetics, which indicates that it will accumulate in different peripheral compartments with different affinities and then redistribute to other organs via the central compartment over time. Looking at the individual data points in Figure S2C (Supporting Information) the increase in lung concentration at *t* = 96 h is reproducible in all six animals. Notably, the dosing plan was also randomized so that the results cannot be explained by a group effect. We therefore conclude that the observed effect is sound. Despite the inherent variability in lung dose, it was confirmed that substantial amounts of i.n. instilled BDQ reached the lungs and was retained there over the 96 h study period.

The i.n. administration route was the only study group in which free BDQ was present in the ELF above the MIC up to the 3 h time point (Figure 4D). Quantification of free drug in the ELF and lavaged lung tissue is not performed routinely in all studies but, in this case, can provide indirect insights into the in situ release profile of the encapsulated BDQ from the liposomes while in the lung. For example, elevated levels of BDQ in the ELF may indicate retention of the drug in the liposomes for longer periods of time since the liposomal formulation assists in retaining larger amounts of the hydrophobic BDQ within an aqueous compartment. In contrast, oral or i.v.-administered BDQ is expected to reach the lung compartments primarily as a free drug (since iv administered liposomes are not expected to enter the lung intact) and therefore will accumulate in the ELF in very low concentrations due to its high tissue affinity. Indeed, BDQ concentrations in the ELF were above the LOQ following i.v. and po administration, but these values were low and did not reach the MIC threshold.

**Figure 5:**
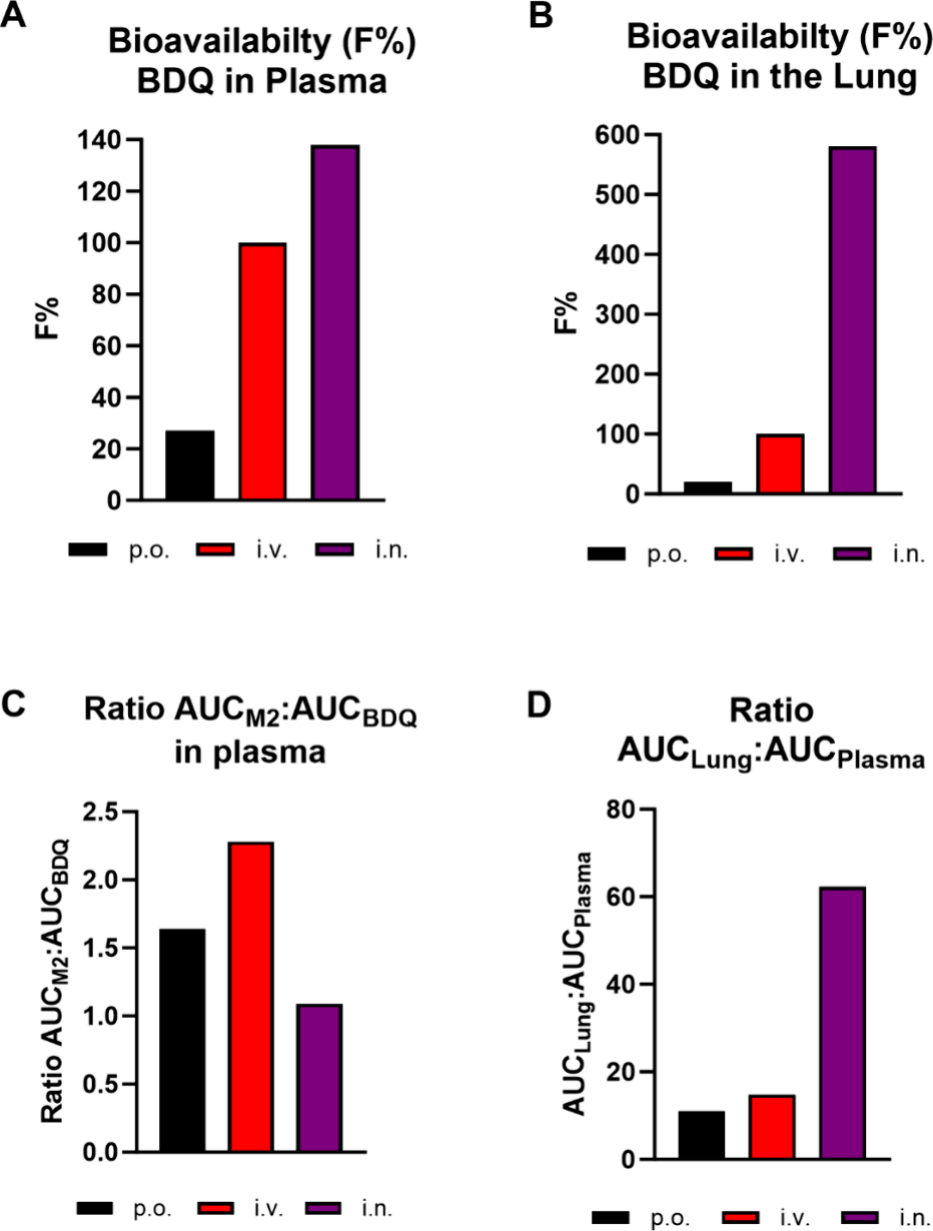
Absolute bioavailability of BDQ in plasma (A) and lung (B) following oral and i.n. administration as compared to i.v. administration. Ratios of AUC_M2_:AUC_BDQ_ in plasma (C) and BDQ AUC_lung_:AUC_plasma_ (D) for each administration route.

The absolute bioavailability of i.n. compared to i.v. administration of BDQ-Lipo_fuc_ formulations was ∼140% in plasma and ∼580% in the lung (Figure 5A,B). The ratio of AUC_M2_:AUC_BDQ_ in plasma was higher after i.v. exposure (ratio = 2.3) compared to i.n. administration (ratio = 1.1) (Figure 5C). It is likely that i.n. administration reduced the first-pass metabolism of BDQ compared to that of oral administration. Information about CYP3A4 expression and activity within the human respiratory tract is controversial. Raunio et al. (2005)^35^ report evidence of CYP isoform expression in the lung, including CY3A4, CYP2C8, and CYP2C19, the major enzymes responsible for BDQ metabolism (Liu et al.^36^), whereas Somers et al. (2007)^37^ found that the CYP3A4 isoform expression was negligible and Phase I activities in the lung were overall <10% compared to the liver.^37^ It is important to note that M2 exposure in mice has been reported to be several-fold higher than in humans;^6^ however, for the purposes of comparing administration routes, we will assume that a reduction in the AUC_M2_:AUC_BDQ_ ratio achieved by intrapulmonary dosing in mice can also translate to humans.

When administered via the oral route, BDQ is known to distribute extensively and accumulate within lung tissue with a reported AUC_Lung_:AUC_plasma_ ratio of ∼20 and 100–200 for BDQ and M2, respectively.^12^ I.n. administration of BDQ-Lipo_fuc_ increased the lung targeting effect of BDQ a further 3-fold with a AUC_Lung_:AUC_plasma_ ratio of 62 (Figure 5D), confirming the hypothesis that i.n. or intrapulmonary administration can achieve higher local lung concentrations of BDQ compared to oral or even i.v. administration. As discussed above, it remains to be determined whether the high BDQ concentrations recovered in lung tissue following i.n. dosing represent a free drug, drug bound to tissue proteins, or drug tightly sequestered within intracellular phospholipid inclusion bodies.^12^ Rouan et al., for example, report that lung tissue concentrations did not always correlate with bactericidal activity, possibly due to tissue binding or sequestration of BDQ and M2 within acidic intracellular compartments.^12^ For this reason, they used plasma data in their PK–PD evaluation as a proxy for the therapeutically active drug fraction. They reported that plasma exposure (AUC) above the MIC value is the primary driver for bactericidal activity of both BDQ and M2, whereby the M2 contribution to activity is only minor. Neither the dosing frequency, *C*_max_ above MIC nor the time above MIC affected the bactericidal activity. In the current study, a combination of liposomal encapsulation and intranasal administration can result in a shift in the PK profile of both BDQ and M2 compared to oral and i.v. administration. However, further studies are required to address the following: (1) whether the liposomes significantly reduce the amounts of tissue-bound or intracellularly sequestered BDQ, thereby resulting in a higher AUC at the site of infection (rather than the plasma), (2) improving or reducing granuloma penetration of BDQ, and (3) altering the dose–response profile in a clinically relevant manner.

## Conclusions

A liposomal formulation for the highly lipophilic drug, BDQ, was administered intranasally to mice, and the pharmacodynamic and pharmacokinetic behavior was examined. The solubilization of BDQ within the liposomal bilayer was hypothesized to provide advantages in terms of both drug bioavailability and local lung tolerance, since the inhalation of high-dose powder formulations of poorly soluble compounds is known to be associated with particle accumulation and side effects, such as cough. In a C3HeB/FeJ murine model of TB infection, the intranasally administered liposomal BDQ formulations (5 mg/kg BDQ, every other day for 2 weeks) achieved a significant reduction in the lung burden of *Mtb* and a higher BDQ concentration in the lung compared to oral or i.v. administration. This promising result justifies further investigation into the therapeutic benefits of inhaled liposomal BDQ. Future studies should focus on inhalation administration in a larger animal model, such as the guinea pig, which will enable nebulization administration of the formulation to the lung, thereby achieving a more realistic drug distribution pattern compared to intranasal delivery. A second advantage of using a larger animal model would be the ability to determine the spatial distribution of BDQ within the lung tissue itself.^27^ Due to their small size and resulting difficulties in quantifying BDQ content in the caseous granuloma of the infected mouse lung,^27^ it is currently unclear whether inhaled BDQ would accumulate primarily in the noninvolved lung tissue or is able to penetrate granulomas in higher quantities compared to oral or i.v. administered BDQ. Yet, despite this open question, the current study demonstrates clearly that i.n. administration resulted in a substantial reduction in the systemic exposure to the M2 metabolite, a compound associated with an elevated risk of QT-prolongation in some patients, thereby supporting the claim that inhaled liposomal BDQ may exhibit clinically relevant activity with a reduced side-effect profile.

## Materials and Methods

### Materials

1,2-Dimyristoyl-*sn*-glycero-3-phosphocholine (DMPC) and 1,2-dimyristoyl-*sn*-glycero-3-phospho-rac-glycerol sodium salt (DMPG-Na) were purchased from Lipoid (Ludwigshafen, Germany), and the fucosylated targeting ligand was provided by Rodos Biotarget GmbH, Hannover, Germany. Bedaquiline fumarate was obtained from MedChemExpress, Monmouth Junction, NJ, USA. *N*-Methyl-bedaquiline (M2) was purchased from TLC Pharmaceutical Standards, Newmarket, ON, Canada. Texas Red 1,2-dihexadecanoyl-*sn*-glycero-3-phosphoethanolamine, triethylammonium salt (Texas Red DHPE) was purchased from Thermo Fisher Scientific, Germany. All solvents were of LC-MS grade, if not stated otherwise.

### Preparation and Characterization of Fucosylated Liposomes

BDQ-loaded and empty fucosylated/nonfucosylated liposomes were prepared via a thin-film hydration method followed by extrusion.^19,20^ Briefly, stock solutions of DMPG-Na dissolved in ethanol/water, DMPC dissolved in chloroform, BDQ dissolved in methanol, and the fucosylated targeting ligand dissolved in ethanol were prepared. The targeting ligand is an amphiphilic cholesterol-fucosyl compound, which is incorporated into the liposomal lipid bilayer with the fucosyl residues pointing outward. Depending on the final liposome composition, different stock solutions were combined in a round-bottom flask, and the solvent was removed using a rotary evaporator. The flask was then transferred to a vacuum desiccator and dried in vacuum for 2 days to remove any residual solvent. The final lipid film contained 8% (mole percent) of fucosyl targeting ligand. Dry films were hydrated with PBS (pH = 7.4) for about 10 min and then briefly sonicated at 35 °C until a homogeneous, milky solution was obtained. The solution was then extruded (30×) through a polycarbonate membrane (Whatman Nucleopore Track-Etched Membrane) with a pore size of 200 nm followed by an extrusion through 50 nm (31×) using a hand-held LiposoFast extruder (AVESTIN Europe GmbH, Mannheim, Germany). Finally, samples were dialyzed (RC membrane, MWCO 12–14 kDa) overnight against PBS (pH = 7.4) to remove nonencapsulated BDQ. For *in vivo* visualization, liposomes were labeled with Texas Red DHPE. The dye was dissolved in a methanol/chloroform (9:1) mixture and added to the flask together with the other stock solutions during lipid film preparation. The final dye content was 0.1% (mole percent).

### Size, PDI, and BDQ Quantification

Hydrodynamic diameters of the BDQ-Lipo_fuc_/BDQ-Lipo formulations were determined by dynamic light scattering (DLS) using a Zetasizer ZS Series instrument (Malvern Instruments Limited, Malvern, UK). BDQ concentrations in the liposomal suspension were established for six independent BDQ-Lipo_fuc_/BDQ-Lipo batches by using LC-MS/MS. A full description of the method is provided in the Supporting Information (ESI). BDQ content per mL liquid nanodispersion was determined and the mean and standard deviation values were calculated.

### *In Vitro* Antitubercular Activity in Infected Murine Bone Marrow-Derived Macrophages

For a detailed description, please refer to the ESI. Briefly, murine bone marrow-derived macrophages (mouse strain C57BL/6 J) were seeded at 1 × 10^5^ cells/well in 48-well plates culture medium (DMEM plus 10% FBS, 100 μg l-glutamine). The cells were incubated with *Mtb* H37Rv (at 37 °C, 5% CO_2_) for 2 h and then washed with a culture medium to remove extracellular mycobacteria. BDQ was administered either as a diluted solution prepared from a DMF stock (20 mg/mL) or as undiluted BDQ-Lipo_fuc_/BDQ-Lipo formulations at BDQ concentrations of 1, 0.1, and 0.01 μg/mL. Samples were incubated for 72 h with infected macrophages, and the number of intracellular bacteria was determined by colony counts after osmotic lysis of the macrophages at *t* = 24, 48, and 72 h incubation (*n* = 3 independent experiments). Vehicle controls were BDQ-free equivalent concentrations of liposomal formulations.

### *In Vivo* Antitubercular Activity Following Intranasal Administration

All experiments were approved by the Ethics Committee for Animal Experiments of the Ministry for Agriculture, Environment and Rural Areas of the State of the Schleswig-Holstein, Germany under license V 244–34653.2016 (63–5/16)/”). Briefly, 6- to 8-week-old female C3HeB/FeJ (Jackson Laboratories, USA) mice were housed in a specific pathogen-free BSL3 lab. C3HeB/FeJ mice were chosen due to their ability to form caseating necrotic granulomas (in contrast to other mouse strains, such as Balb/c), which better represent the human lung response to *Mtb* infection in terms of granuloma formation and higher resistance to drug therapy.^26,27^ Animals were infected with the virulent (H37Rv) strain of *Mtb* using the aerosol route at day 0. At 30 days postinfection, mice were separated into five treatment groups (Table 3) and given a total of seven administrations of either BDQ-loaded liposomes (fucosylated/nonfucosylated) or controls. Negative controls consisted of untreated animals, while the “positive control” consisted of solubilized BDQ (vehicle: sterile acidified 20% HPCD; 3.6 mg/mL BDQ; pH 3). Dosing was performed every second day for 2 weeks. Intranasal (i.n.) instillation of 20 μL sample per nostril was used as a minimally invasive and material-sparing method to achieve upper and lower respiratory tract delivery. In samples with drug or liposomes, this volume contained 160 μg of BDQ per 40 μL dose (5 mg/kg) and ∼2.5 mg total lipids. Texas Red-labeled phosphatidyl ethanolamine was used to confirm the presence of the formulation in the lung following i.n. administration.

**Table 3:** Treatment Groups for In Vivo Efficacy Studies

#	treatment	route	dose (mg/kg)	BDQ conc. (mg/mL)	lipid conc. (mg/mL)	*N*
1	untreated		0	0	0	6
2	Lipo_fuc_ (no BDQ)	i.n.	0	0	64	6
3	BDQ solution (HPCD)	i.n.	5	4	0	6
4	BDQ-Lipo (no fucosylation)	i.n.	5	4	64	6
5	BDQ-Lipo_fuc_	i.n.	5	4	64	6

After animals were sacrificed, bacterial burdens in the lungs and spleen were determined. Whole organs were harvested, weighed, and mechanically ground in 1 mL of WTA (water:Tween 80 at 0.01%: albumin at 0.05%) buffer inside a Whirpak plastic bag using a 50 mL Falcon tube and Petri dish. Organ homogenates were serially 10-fold diluted in WTA buffer and 100 μL were plated onto Middlebrook 7H11 agar plates using glass rods and incubated at 37 °C. After 21–28 days, mycobacterial colonies were counted. Δlog_10_ CFU values were calculated by subtracting the mean log_10_ CFU of the treatment group from the mean log_10_ CFU of the untreated controls. Individual log_10_ CFU values from Irwin et al. (2016)^27^ were extracted from the manuscript graphs using Web Plot Digitizer (Version 4.6), distributed under the GNU Affero General Public License Version 3, copyright 2010–2022 Ankit Rohatgi (ankitrohatgi@hotmail.com).

### *In Vivo* Pharmacokinetics Following Intranasal, Intravenous, and Oral Administration

All experiments were approved by the Ethics Committee for Animal Experiments of the Ministry for Consumer Protection and Veterinary Affairs, State of Saxony-Anhalt, Germany, under the license 203.m-42502–2–1632 MLU G. For a detailed description of the methodology please refer to the ESI. Nine- to 11-week-old male and female Balb/c mice (Charles River, Germany) were used for all pharmacokinetic studies. Three administration routes were compared: intravenous (i.v.; BDQ-Lipo_fuc_), intranasal (i.n.; BDQ-Lipo_fuc_), and oral (p.o.; neat BDQ). Six animals per time point (0.5, 3, 24, 48, 72, and 96 h) were used. For i.v. administration, a single bolus injection (100 μL; 2.5 mg/kg) of the BDQ-Lipo_fuc_ formulation was injected into the lateral tail vein. For i.n. administration, animals were lightly anesthetized with 2.5% inhaled isoflurane (in O_2_; at 3 L/min), and 50 μL of the BDQ-Lipo_fuc_ formulation was added to each nostril sequentially. (2 × 50 μL; 2.5 mg/kg). For both i.v. and i.n. administration, the BDQ-Lipo_fuc_ formulations were diluted in sterile PBS prior to administration. Oral administration of the neat BDQ (powder suspended in 5% glucose containing 1% hydroxypropylmethyl cellulose; 200 μL; 25 mg/kg) was performed by gavage using soft, sterile polypropylene dosing probes (Instech, Germany) according to the manufacturer’s instructions without anesthesia. To avoid group effects, administration of the test substances was randomized and conducted over 3 weeks. Each week, *n* = 2 (one male and one female) animals from each treatment group and time point were administered test substances. At the designated time points, animals were euthanized by a low CO_2_ flow rate^38^ followed immediately by terminal cardiac puncture. Blood samples were collected in prelabeled tubes containing anticoagulants (0.109 M sodium citrate).

### LC-MS/MS Quantification of BDQ and *N*-Desmethyl-bedaquiline (M2) of PK Samples

For a detailed description of the methodology, please refer to the ESI. Calibration curves ranging from 0.00025 to 0.250 μg/mL were prepared for BDQ and M2 in plasma and additionally for BDQ in extracts from lung tissue homogenate, BAL, and the cellular fraction of the BAL. Table 4 lists the limit of detection (LOD) and limit of quantification (LOQ) values for each compound in each compartment. Sample extracts (prepared in the same manner as the calibration curves and quality controls) were analyzed by LC-MS/MS using a triple-quadrupole mass spectrometer XEVO TQ-MS (Waters, Milford, MA, USA) coupled to a high-performance liquid chromatography setup (Agilent 1200, Agilent, Santa Clara, CA, USA).

**Table 4:** LOD and LOQ Values for BDQ/M2 in the Compartments Tested

compound	compartment	LOD	LOQ
BDQ	plasma	0.009 μg/mL	0.028 μg/mL
M2	plasma	0.002 μg/mL	0.010 μg/mL
BDQ	lung tissue	0.016 μg/g	0.050 μg/g
BDQ	ELF	0.004 μg/mL	0.013 μg/mL

### Calculation of Noncompartmental PK Parameters

The harvesting of ELF and lung tissue at each time point are terminal end points; therefore, it should be noted that concentration–time profiles could not be calculated for single animals. Instead, mean BDQ concentrations, calculated from *n* = 6 animals per administration route, compartment, and time point, were plotted against time. The maximal concentration (*C*_max_) and time (*t*_max_) were estimated from the concentration–time curves without fitting into a model. The area under the curve from *t* = 0–96 h (AUC_*t*=0–96 h_) was calculated using GraphPad Prism software (v9.4.1) setting the LOQ values for each compound/compartment as the baseline. The absolute bioavailability was calculated in plasma for both BDQ and M2, whereby the i.v.-administered BDQ-Lipo_fuc_ formulation served as the reference group. Additionally, the ratio of BDQ:M2 AUCs in plasma as well as the ratio of the AUC lung:plasma were calculated for each administration route. Further details can be found in the ESI.

### Statistical Analysis

One-way ANOVA with a posthoc Bonferroni correction was performed using GraphPad Prism (v10.0) to compare multiple data sets. Significance was defined as *p* < 0.05.

## Supporting Information Available

Detailed materials and methods, distribution of Texas red-labelled PE present in BDQ-Lipofuc systems in lung tissue slices taken from one representative C3HeB/FeJ mouse that had been administered seven i.n. doses of BDQ-Lipofuc (5 mg/kg) every second day for 2 weeks, and pharmacokinetic data from Figure 5 as individual values to show the data variation observed in the study (PDF)
