# Supplementary material for: Intranasal Administration of Bedaquiline-Loaded Fucosylated Liposomes Provides Anti-Tubercular Activity while Reducing the Potential for Systemic Side Effects
Source: ACS Infect Dis. 2024 Aug 13;10(9):3222–32. doi: 10.1021/acsinfecdis.4c00192 (PMC11406518; doi:10.1021/acsinfecdis.4c00192)
Supplement: Supplementary file 1 — id4c00192_si_001.pdf [file id4c00192_si_001.pdf]

Supporting Information

Intranasal administration of bedaquiline-loaded fucosylated liposomes provides anti-tubercular activity while reducing the potential for systemic side-effects

Franziska Marwitz[1,2], Gabriela Hädrich[3,4], Natalja Redinger[5], Karen F. W. Besecke[6,7,8], Feng Li[3,4,9], Nadine Aboutara[1,2], Simone Thomsen[1], Michaela Cohrs[4,10], Paul Robert Neumann[4], Henrike Lucas[4], Julia Kollan[4], Constantin Hozsa[6,7], Robert K. Gieseler[6,11], Dominik Schwudke[1,2,12,13], Marcus Furch[6,14], Ulrich Schaible[2,5], Lea Ann Dailey[3]*

[1] Bioanalytical Chemistry, Research Center Borstel, Leibniz Lung Center, Parkallee 1–40, 23845 Borstel, Germany.
[2] German Center for Infection Research, Thematic Translational Unit Tuberculosis, 23845 Borstel, Germany;
[3] Department of Pharmaceutical Sciences, University of Vienna, Josef-Holaubek-Platz 2, 1090 Vienna, Austria
[4] Institute of Pharmacy, Martin-Luther-Universität Halle-Wittenberg, Kurt-Mothes-Str. 3, 06120 Halle/Saale, Germany
[5] Cellular Microbiology, Research Center Borstel, Leibniz Lung Center, Parkallee 1–40, 23845 Borstel, Germany.
[6] Rodos Biotarget GmbH, Feodor-Lynen-Straße 31, 30625 Hannover, Germany
[7] Siegfried Hameln GmbH, Langes Feld 13, 31789 Hameln, Germany
[8] Cardior Pharmaceuticals GmbH, Hollerithallee 20, 30419 Hannover, Germany
[9] Vienna Doctoral School of Pharmaceutical, Nutritional and Sport Sciences (PhaNuSpo), University of Vienna, Josef-Holaubek-Platz 2, 1090 Vienna, Austria
[10] General Biochemistry and Physical Pharmacy, Faculty of Pharmaceutical Sciences, Ghent University, Ottergemsesteenweg 460, 9000 Ghent, Belgium
[11] Department of Medicine, University Hospital, Knappschaftskrankenhaus Bochum, Ruhr University Bochum, In der Schornau 23–25, 44892 Bochum, Germany
[12] German Center for Lung Research (DZL), Airway Research Center North (ARCN), Research Center Borstel, Leibniz Lung Center, 23845 Borstel, Germany
[13] Kiel Nano, Surface and Interface Sciences (KiNSIS), Kiel University, 24118 Kiel, Germany
[14] Certmedica International GmbH, Magnolienweg 17, 63741 Aschaffenburg, Germany

*Corresponding author: leaann.dailey@univie.ac.at

F. Marwitz, G. Hädrich and N. Redinger contributed equally to the study.

Detailed Materials and Methods

BDQ quantification in formulations: BDQ and total lipid concentrations were established for ten independent BDQ-LNCfuc batches. Liquid chromatography was performed on an Agilent 1100 Series HPLC (Agilent Technologies, Santa Clara, CA) using a SeQuant ZIC-HILIC column (Merck Millipore SeQuant, 2.1 inner diameter × 150 mm length with 5 µm particle size, pore size 200 Å) at a column temperature of 30 °C. The mobile phase consisted of 1 % formic acid (FA, solvent A) and acetonitrile (ACN, solvent B). 20 µL of BDQ-loaded fucosylated liposomes were diluted in 800 µL ACN and 180 µL 1 % FA, followed by thorough vortexing. Afterwards, 20 µL of this mixture were further diluted in 800 µL ACN and 180 µL 1 % FA. The solution was vortexed again and then centrifuged for 10 min at 15.000 × g at RT. ≈500 µL of the resulting supernatant were transferred to a 1.5 mL Eppendorf tube and recentrifuged under the same conditions. Next, 60 µL supernatant were transferred to a vial (three aliquots per sample); the injection volume was 5 µL for LC-MS/MS analysis.

Analysis was performed using an Accela UHPLC system coupled with a TSQ Quantum Access Max tandem quadrupole mass spectrometer (both from Thermo Fisher Scientific, Waltham, USA). An Accucore RP-MS column (150 × 2.1 mm, 1.7 µm; Thermo Fisher Scientific) was eluted with mobile phase A consisting of acetonitrile + 0.1 % formic acid and mobile phase B consisting of H_2O + 0.1 % formic acid. The gradient elution was applied as follows: 2 min elution at 10 % A and a subsequent increase to 99 % A until minute 8, which was then maintained for 3 min, followed by a return to initial conditions. The system was operated at a flow rate of 0.3 mL min^{-1}, with the column oven set at 40 °C. The transitions were monitored using selected reaction monitoring of BDQ (parent mass 557.15 m/z) with fragments A (58.2 m/z) and B (330.06 m/z) using heated electrospray ionization (H-ESI) in the positive ion mode. The entire system was operated via the standard software Xcalibur (Thermo Fisher Scientific).

In vitro antitubercular activity assay in infected macrophages

All studies were performed in a biosafety lab category 3 (BSL3). Murine bone marrow-derived macrophages (mouse strain C57BL/6 J) were seeded at 1 × 10^5 cells/well in 48-well plates in culture medium (DMEM plus 10 % FBS, 100 µg L-glutamine). The cells were incubated with *M. tuberculosis* H37Rv (at 37 ^{0}C, 5 % CO_2) for 2 h and then washed with culture medium to remove extracellular mycobacteria. The Multiplicity of Infection (MOI) was 2:1 (mycobacteria: macrophage). BDQ was administered either as a diluted solution prepared from a DMF stock (non-formulated control) or as undiluted BDQ-$Lipo_{fuc}$/BDQ-Lipo formulations. Samples were incubated for 72 h with infected macrophages using BDQ doses of 1, 0.1, or 0.01 µg/mL, respectively. Vehicle controls were BDQ-free equivalent concentrations of unloaded liposomal formulations. Numbers of intracellular bacteria were determined by colony counts after osmotic lysis of the macrophages after t=24, 48 and 72 h incubation (n=3 independent experiments).

In vivo antitubercular activity following intranasal administration

Six to eight-week-old female C3HeB/FeJ mice (Jackson Laboratories, USA) were used for the experiments. All animals were housed in individually ventilated cages containing filters in a

specific pathogen-free BSL3 lab according to the German animal protection law and approved by the Ethics Committee for Animal Experiments of the Ministry for Agriculture, Environment and Rural Areas of the State of the Schleswig-Holstein, Germany under the license V 244-34653.2016 (63-5/16), entitled *"Analyse von Anti-Tuberkulosemitteln in Verbindung mit neuen Nanopartikel-basierten Wirkstoffträgern (mittels bildgebender Verfahren)" oder Kurzbezeichnung "Nanopartikel-basierte Wirkstoffe gegen die Tuberkulose"* – i.e., "Analysis of anti-tuberculosis agents in combination with new nanoparticle-based drug carriers (using imaging techniques)" or briefly "Nanoparticle-based agents against tuberculosis".

Animals were infected with a virulent strain (H37Rv) of *M. tuberculosis* using the aerosol route at day 0. Previously prepared frozen *M. tuberculosis* stock was thawed and resuspended using a 1 mL syringe with a 27G3/4 cannula to produce a uniform distribution of mycobacteria in a suspension. The bacterial suspension was diluted in sterile distilled water to a volume of 6 mL, of which 5.5 mL was nebulized such that each animal received 100 mycobacteria. The remaining 500 µL of the suspension was serially diluted and plated in Mycobacteria 7H11 agar plates and incubated for 3-4 weeks at 37°C to confirm viability. For aerosol infection, mice were placed inside a specialized metal cage inside the aerosol chamber (GlasCol), and 5.5 mL of the bacterial suspension was nebulised into the aerosol chamber. Inside the chamber, animals were exposed to low-dose *M. tuberculosis* aerosol by regulating both the mainstream air flow (~1.68 m^3/h) and the compressed air flow (~0.28 m^3/h) during nebulization.

At 30 days post-infection, mice were distributed to five treatment groups for seven administrations, i.e., once every second day for two weeks. Intranasal (i.n.) instillation of a 40 µL sample was used as a minimally invasive and material-sparing method to achieve upper and lower respiratory tract delivery. In samples containing active agent, this volume contained 160 µg BDQ per dose (5 mg/kg) and ~2.5 mg of total lipids. Southam et al [Southam] have demonstrated that nasal instillation of volumes >35 µL results in high lung concentrations of labelled molecules and colloids. Based on this data, it would be expected that approximately 50 % of a 40 µL i.n. instillation volume would reach the lungs, with the remaining amount located predominantly in the nasal cavities (thus avoiding oral absorption).

After animals were sacrificed, bacterial burdens in the lungs and spleen were evaluated. Whole organs were harvested, weighed and mechanically ground in 1 mL WTA (water: Tween-80 (0.01 %): albumin (0.05 %)) buffer inside a Whirpak plastic bag using a 50 mL Falcon tube and Petri dish. Organ homogenates were serially 10-fold diluted in WTA buffer and 100 µL were plated onto Middlebrook 7H11 agar plates using glass rods and incubated at 37°C. After 21-28 days, mycobacterial colonies were counted.

In vivo **pharmacokinetics following intranasal, intravenous and oral administration**

Nine- to eleven-week-old male and female Balb/c mice (Charles River, Germany) were used for all pharmacokinetic studies. All experiments were performed in line with the German animal protection law and approved by the Ethics Committee for Animal Experiments of the Ministry for Consumer Protection and Veterinary Affairs, State of Saxony-Anhalt, Germany, under the license 203.m-42502-2-1632 MLU G, entitled *„Bestimmung der pharmakokinetischen Parameter von Antibiotika-beladenen Nanocarriern nach intravenöser und inhalativer Gabe"* – i.e.,

"Determination of the pharmacokinetic parameters of antibiotic-loaded nanocarriers after intravenous and inhalation administration".

Three administration routes were compared: intravenous (i.v.; BDQ-LNC_{fuc}), intranasal (i.n.; BDQ-LNC_{fuc}) and oral (p.o.; neat BDQ). Six animals per time point (0.5, 3, 24, 48, 72, 96 h) were used. For i.v. administration, a single bolus injection (100 µL; 2.5 mg/kg) of the BDQ-LNC_{fuc} formulation was injected into the lateral tail vein using a 27G needle. Prior to i.n. administration, animals were lightly anesthetized with 2.5 % inhaled isoflurane (in O_2; at 3 L/min). One nostril was closed while 50 µL of the BDQ-LNC_{fuc} formulation was added as a drop into the open nostril and inhaled. Approximately 30 s thereafter, this procedure was repeated for the other nostril (2 x 50 µL; 2.5 mg/kg). For both i.v. and i.n. administration, the BDQ-LNC_{fuc} formulations were diluted in sterile PBS prior to administration. Oral administration of the neat BDQ (powder suspended in 5 % glucose containing 1 % hydroxypropylmethylcellulose; 200 µL; 25 mg/kg) was performed by gavage using soft, sterile polypropylene dosing probes (Instech, Germany) according to the manufacturer's instructions without anesthesia. At the designated time points, animals were euthanized by cervical dislocation followed immediately by terminal cardiac puncture. Blood samples were collected in pre-labelled tubes containing anticoagulant (0.109 M sodium citrate).

Sample preparation for Quantification of BDQ and N-desmethyl-bedaquiline (M2)

Commercially available human citrate plasma (Sigma Plasma, Sigma Aldrich, St. Louis, MO, USA) was used for preparation of the calibration curve and quality control samples (QC). Analyte-free plasma (5 µL) was diluted 1:10 with water (LICHROSOLV®, SUPELCO, Darmstadt, Germany) and subsequently mixed with 150 µL consisting of 98 % acetonitrile (ACN) (HiPerSolv CHROMANORM®, VWR chemicals, Radnor, PA USA) and 2 % formic acid (diluted to 1 %, ROTIPURAN® ≥99 %, Roth, Karlsruhe, Germany) containing the internal standard (ISTD) reserpine (Sigma Aldrich) in an Eppendorf tube (1.5 mL SafeLockTube, Eppendorf, Hamburg Germany) giving an end volume of 200 µL.

After 30 s of vortexing (IKA MS2 Minishaker, IKA, Staufen, Germany) the samples were shaken for 10 min with 1,300 rpm at RT (NeoMixCool, neolab Migge, Heidelberg, Germany) and afterwards centrifuged at 15,000 *g* for 10 min at 18°C (Centrifuge 5804R, Eppendorf); 190 µL of the supernatant was then transferred to a new tube. Standard solutions (10 µL) containing both BDQ (Adooq Bioscience, Irvine, CA, USA) and M2 (TLC pharmaceutical standards, Newmarket, ON, Canada) were added to the extracted blank plasma to reach the desired concentration. Study samples were extracted in the same manner, whereby the concentrations of the blank plasma extract and a dilution factor of 1:4 were accounted for. Calibration curves covered a range of 0.00025, 0.001, 0.004, 0.016, 0.0063, and 0.250 µg/mL, and the QC standard concentration was 0.00063 µg/mL. Solutions were pipetted into glass vials (9 mm short thread screw, with 300 µL insert, Wicom, Heppenheim, Germany) with PTFE caps (0,25 mm, Machery Nagel, Düren, Germany) for analysis by LC-MS/MS. All samples were measured with technical duplicates.

LC-MS/MS method for drug quantitation in biological sample matrices

Quantification was performed using a tandem-quadrupole mass spectrometer XEVO TQ-MS (Waters, Milford, MA, USA) coupled to a high-performance liquid chromatography set-up (Agilent

1200, Agilent, Santa Clara, CA, USA) via the ESI - source. For controlling system operation parameters and to acquire and process data, the software Masslynx 4.1 (Waters) was used. LC separation was achieved using a CSH C18 5µm, 2.1 x 150 mm column (X Select, Waters) with eluent A (water with 1 % formic acid (ROTIPURAN® ≥99 %)) and B (ACN with 1 % formic acid) following a gradient: start with 90 % A, 2 min 60 % A, 5 min 50 % A, 9 min 20 % A, 10 min 10 % A, 12 min 90 % A. The total run-time per sample was 14 min and the flow rate was 0.5 mL/min. Column temperature was set to 30°C. The samples were cooled at 4 °C until injection in the autosampler. A post-column split was applied resulting in a flow of approx. 100 µL/min at the ESI source. MS parameters were as follows: source temperature: 120 °C, desolvation temperature: 350 °C, desolvation gas flow 350 L/h, capillary voltage: 3 kV. For each analyte and the ISTD, two m/z-transitions were chosen. For BDQ (556.96 > 58.08 and 556.96 > 228.91) and N-desmethyl bedaquiline (M2) (542.90 > 467.75 and 542.90 > 481.72), a cone voltage of 20 V and a collision energy of 25 eV was chosen for both transitions, while for reserpine (608.86 > 173.85 and 608.86 > 194.96) were used for 50 V and 30 eV, respectively.

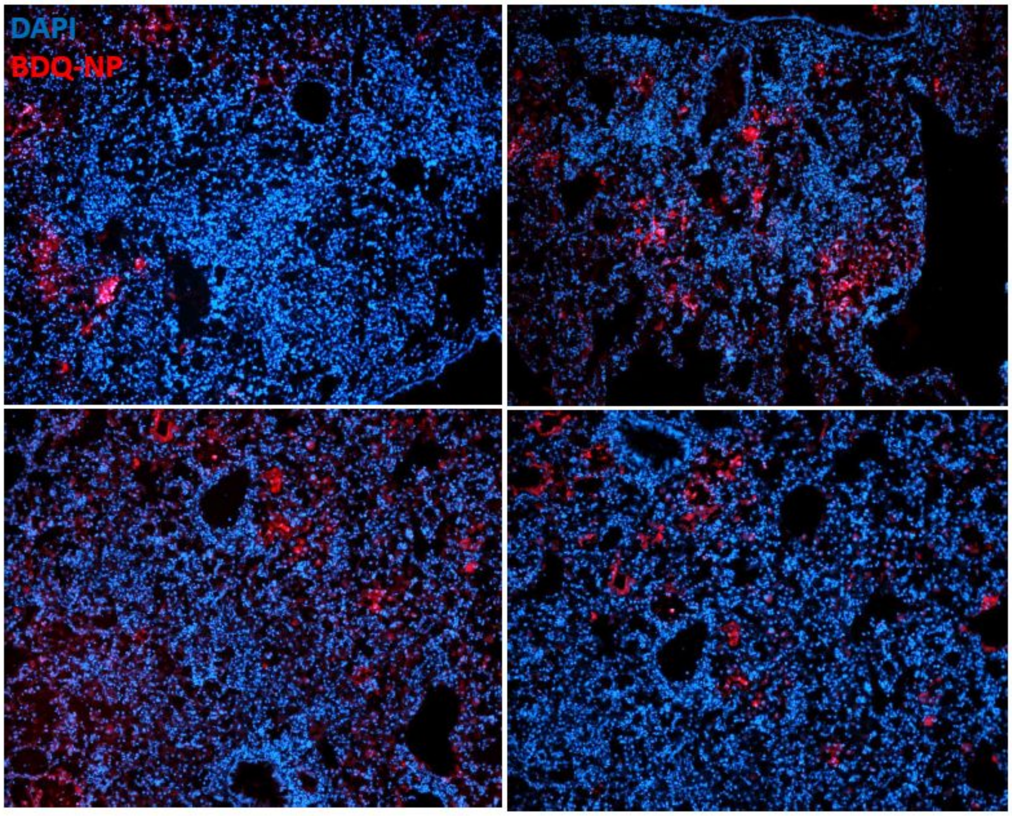

***Figure S1**. Distribution of Texas red-labelled PE present in BDQ-Lipofuc systems in lung tissue slices taken from one representative C3HeB/FeJ mouse that had been administered seven i.n. doses of BDQ-Lipofuc (5 mg/kg) every second day for two weeks (day 44 post-infection).*

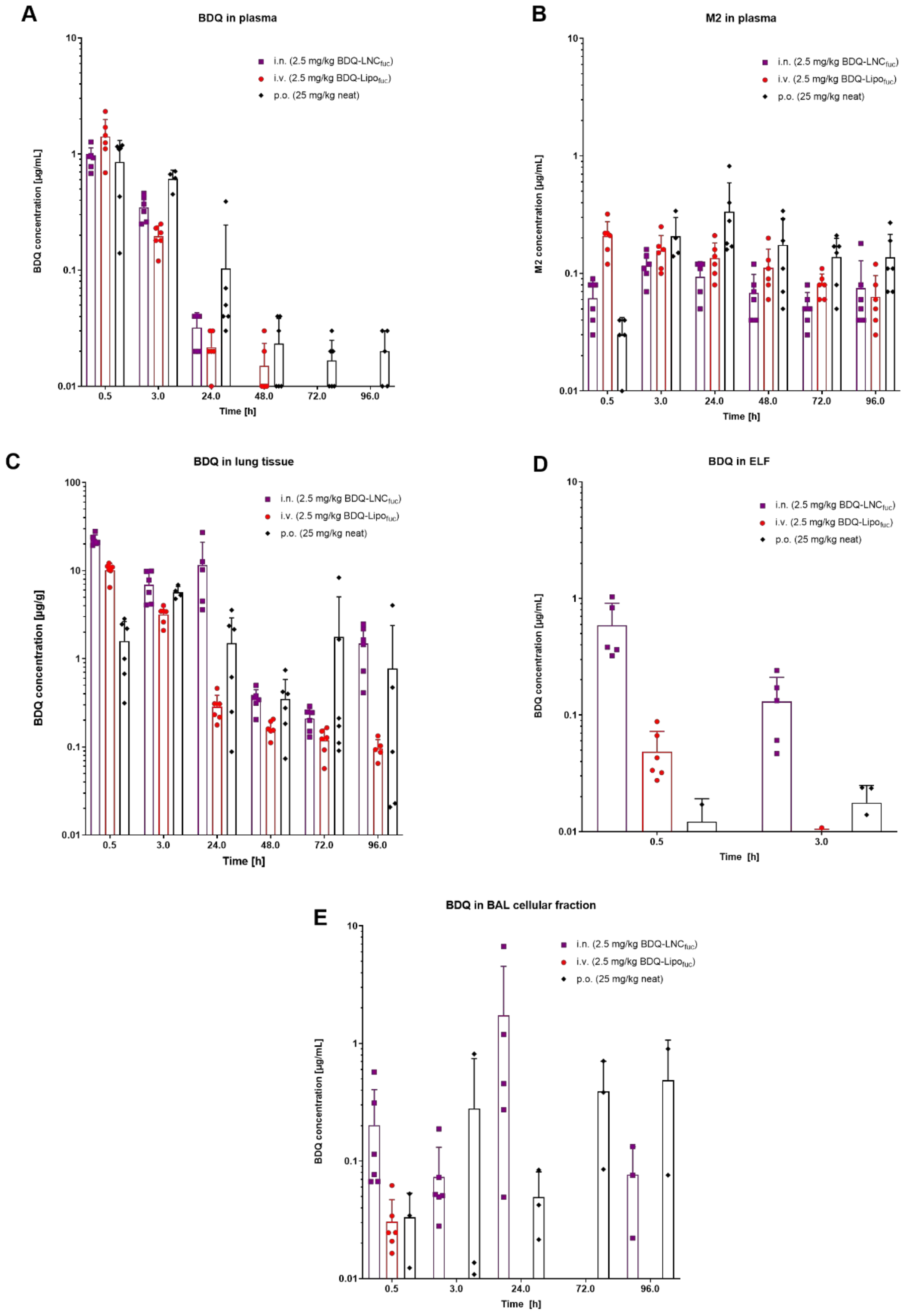

Figure S2. *The pharmacokinetic data from Figure 5 is plotted here as individual values to show the data variation observed in the study. The bars represent mean and standard deviation from the individual data points in each group at each time point.*
